# Prospective effect of mesenchymal exosomes versus nanocurcumin-loaded mesenchymal exosomes on induced periodontitis in albino rats

**DOI:** 10.1007/s10266-025-01161-x

**Published:** 2025-08-03

**Authors:** Nehad M. Abd-elmonsif, Sherif Gamal, Mona El Deeb, Amany A. Rabea

**Affiliations:** 1 Department of Oral Biology, Faculty of Oral and Dental Medicine, Future University in Egypt, Cairo, Egypt; 2 Research Labs Supervisor, Faculty of Pharmacy, Future University in Egypt, Cairo, Egypt

**Keywords:** Periodontitis, Exosomes, Nanocurcumin, Nanocurcumin-loaded mesenchymal exosomes

## Abstract

Provide insights on the effect of mesenchymal exosomes and Nanocurcumin (NCUR)-loaded exosomes on periodontitis. To induce periodontitis, 42 rats were injected with 3 µL of a 10 mg/mL lipopolysaccharide (LPS) for 4 weeks and divided into 3 categories: untreated Periodontitis, Exosomes treated (single dose 200 µg exosomes), and exosomes loaded NCUR treated group (200 µg exosomes loaded with 200 µg NCUR). In addition, 14 rats were injected PBS to serve as control. Rats were sacrificed between 2 and 4 weeks. Rats were allocated in 7 groups; (Group I (Control), Group II (Periodontitis 2 weeks), Group III (Periodontitis + exosomes 2 weeks), Group IV (Periodontitis + loaded exosomes 2 weeks), Group V (Periodontitis 4 weeks), Group VI (Periodontitis + exosomes 4 weeks) and Group VII (Periodontitis + loaded exosomes 4 weeks). The specimens were prepared for histological, histochemical and ELISA analysis. Histological examination of Group I showed normal structure of periodontium. Groups II and V illustrated reduction in periodontal ligament and different stainability of cementum and bone. Group III revealed disordered periodontal fibers and irregular outlines of cementum and bone. The periodontal fibers in Groups IV and VII were obliquely oriented, and the cementum and bone were consistently stained. Group VI explored dense periodontal ligament. Cementum and alveolar bone showed regular outlines. Masson's trichrome statistical results showed group VI has highest mean. Group V has the greatest IL-1β mean. NCUR-loaded exosomes were found to be more effective in decreasing inflammation and stimulating tissue regeneration in experimental periodontitis.

## Introduction

Periodontitis is a considerable global health issue of high level of occurrence. This inflammatory condition is brought on by local microbial imbalance, and it is the main factor causing the periodontium to permanently deteriorate [1]. Microflorae secrete lipopolysaccharides (LPS), which are thought to be a main contributor in pathogenesis of periodontitis via stimulation of pro-inflammatory mediators [2, 3]. In many clinical scenarios, current periodontitis therapeutic strategies are useful in decreasing or preventing the pathogenic state, but they are not consistently successful in restoring the lost periodontal structures [1].

Nanotechnology has brought significant advancements to the field of dentistry. The incorporation of nanostructured surfaces in dental implants has been shown to enhance osseointegration and overall implant stability. In restorative dentistry, nanoparticles contribute to improved composite and adhesive strength, as well as enhanced aesthetics and durability. In addition, nanotechnology has found applications across various dental disciplines, including orthodontics, prosthodontics, and surgical procedures. Nanodentistry also offers novel avenues for dental therapies, such as management of periodontal disorders and alveolar bone loss [4]. Curcumin nanoparticles (NCUR) are one of these treatment techniques which possess potent antioxidant and anti-inflammatory features that potentially cure periodontitis [5].

In tissue engineering therapy, exosomes produced from mesenchymal stem cells (MSCs-Exo) are of great interest as a cell-free-based therapeutic option [6, 7]. They are secreted by cells as bi-lipid membrane vesicles. When exosomes reach the target cells, their payload of proteins, lipids, and nucleic acids elicit biological reactions that are indicative of the contents of the cargo [8]. Thus, MSCs-Exo serve as a primary mediator of the therapeutic action of MSCs avoiding the contentious concerns associated with using stem cells directly, such as instability, tumorigenicity, severe immune rejection and low survival rate [9]. It is considerable to mention that exosomes exhibit a high degree of immunogenicity and endocytosis, rendering them appropriate natural drug delivery vehicles for therapeutic and clinical uses [10]. Accordingly, they enable effective cell internalization and shield their contents from enzymatic destruction [11]. Moreover, it has been suggested that exosomes released by bone marrow mesenchymal stem cells (BMSC-Exo) offer an alternative viewpoint and a possible treatment strategy for management of periodontitis and alveolar bone resorption [12]. Loading of BMSC-Exo by NCUR “NCUR-encapsulated exosomes” makes NCUR highly bioavailable, soluble, and capable of reaching high blood concentrations without causing toxicity. Therefore, exosomes may be an efficient synthetic nanoparticle transporter of NCUR than other nanoparticles [10].

Nanocurcumin increases curcumin bioavailability [13], whereas exosomes promote targeted delivery and cellular uptake [9]. Their combination is thought to enhance therapeutic outcomes in inflammatory diseases including periodontitis [10]. Therefore, the herein study is designed to compare the histological, histochemical and functional effect of BMSC-Exo versus NCUR-loaded BMSC-Exo on treatment of LPS-induced periodontitis in rat model.

## Materials and methods

### Study sample

56 mature healthy male albino rats, with ages 4–5 weeks, were used in this study. The animals were obtained from the Future University of Egypt's Faculty of Pharmacy's animal house. The animals were kept in pathogen-free cages with regulated humidity and temperature, a cycle of 12-h light and darkness, and the appropriate kind of food and water. The research followed the ARRIVE guidelines to report animal studies. Future University in Egypt's Research Ethics Committee gave its approval to the study (FUE.REC (5)/1–2025).

### Sample size calculation

Sample size, statistical calculator based on 95% confidence interval (CI), and study power of 80% with 5% α error were calculated using MedCalc® statistical software version 12.3.0.0 (MedCalc® software, Ostend, Belgium). A total sample size of 14 (per group) plus an extra 14 samples in the control group enabled the rejection of the null hypothesis, based upon the results of previous study [14].

### Study design

42 rats were given an injection of LPS (dosed at 3 µL of a 10 mg/mL LPS solution) intraperiodontally adjacent to their bilateral lower first molars to induce periodontitis. For 4 weeks, a trained operator administered the injection three times per week using Hamilton-type microsyringes with specially designed needles measuring 30 Ga and 0.6 cm in length (Hamilton Robotics–Agilent Technologies) [15] then the rats were randomized blindly using a random number generator to one of the three groups (14 rats each): untreated periodontitis, exosomes treated group (single dose 200 µg exosomes dissolved in PBS) and exosomes loaded NCUR treated group (single dose 200 µg exosomes loaded with 200 µg NCUR dissolved in PBS) injected intraperiodontally at the same anatomical regions [16]. Control group (14 rats) injected the same volume of PBS in same anatomical region.

Rats were slaughtered by cervical dislocation under dissociative anesthesia 2 and 4 weeks after the end of the experiment. The first molar regions were meticulously resected. The soft tissue was removed for the ELISA analysis, and the hard tissues from the site of injection were obtained out and frozen in liquid nitrogen immediately. They were then stored at −80 °C until they were demanded. For histological analysis, resected areas of first molars were fixed in 4% paraformaldehyde for 24 h at 4 °C until used.

The groups are named as the following: Group I (Control group), Group II (Periodontitis 2 weeks), Group III (Periodontitis + exosomes 2 weeks), Group IV (Periodontitis + loaded exosomes 2 weeks), Group V (Periodontitis 4 weeks), Group VI (Periodontitis + exosomes 4 weeks) and Group VII (Periodontitis + loaded exosomes 4 weeks).

All groups are subjected to:

- Histological and histochemical evaluation of periodontal tissues to evaluate structural changes.
- Quantitative measurement of IL-1β levels using ELISA.
- Quantitative assessment of mean area fraction for newly formed collagen fibers using image analysis for histochemical staining (Masson’s trichrome) sections.

Allocation to groups was performed using a random number generator, and outcome assessors were blinded to the treatment groups throughout histological and ELISA analyses.

### Chemicals and reagents

Escherichia coli LPS (coli O111:B4) and PBS were purchased from Sigma-Aldrich Chemical Co., St. Louis, Mo, U.S.A. NCUR was purchased from Nanotech Egypt for photo-electronics (Al Giza, Egypt).

### MSC-derived exosomes isolation

Exosomes isolation was obtained from rat BM-MSCs line previously prepared at General Histology Unit, Faculty of Medicine, Cairo University.

Exosomes were extracted from the supernatants of BM-MSCs (5 × 106 cells/ml) in their third passage, which were cultivated at Roswell Park Memorial Institute medium (RPMI 1640 medium) and supplemented with 0.5% bovine serum albumin (Sigma® Chemical Co., St. Louis, MO, USA). For 20 min, the cells were centrifuged at 300 g for 10 min, 2,000×g for 10 min, and 10,000×g for 30 min. Using a Beckman Coulter®, Optima L 90 K, USA, ultracentrifuge, the cell-free supernatants were centrifuged at 110, 000×g for 70 min. This was followed by a wash in serum-free medium 199, which included 25 mmol of 4-(2-hydroxyl ethyl)-1-piperazine ethane sulfonic acid (Sigma® Chemical Co., St. Louis, MO, USA). Following that, they underwent ultracentrifugation once more in the identical circumstances. Overnight, the pure exosomes were cultivated in the exosome collection medium [17].

### Characterization of exosomes

Transmission electron microscopy was used to investigate the morphology of exosomes. Formvar/carbon-coated grids were coated with 10 µL of the suspension, which was then allowed to dry at room temperature. A transmission electron microscope (JEOL JEM® 1010, Jeol Ltd, Japan) operated at 120 kV was used to observe the exosome-containing grids after the exosomes had been fixed in 2% glutaraldehyde for 5 min and stained with 4% uranyl acetate.

### Loading of nanocurcumin

200 μg NCUR: 200 μg of exosomes mixture was diluted with PBS then six 30-s on/off cycles lasting 3 min each, with a 2-min cooling interval in between, were sonicated [11] using (Branson Model 3510 Ultrasonic Cleaner, UK) sonicator.

### Characterization of Curcumin nanoparticle loaded on exosomes

#### UV–visible spectroscopy

For confirmation of NCUR loading into the exosomes, the produced NCUR-exosome sample was analyzed using UV–visible spectroscopy [18]. The UV–visible spectrum of NCUR and NCUR-exosomes were measured and compared.

### Evaluation of histological changes and histochemistry assay

The anatomical areas of the previously mentioned groups were collected and fixed with 4% paraformaldehyde and embedded in paraffin and then cut into 4 μm sections.

The sections were deparaffinized and stained with:

- Hematoxylin and eosin (H&E) for routine histological analysis.
- Masson’s trichrome stain to detect the immature collagen (newly formed) (blue color) and mature collagen (old) (red color) [19]. Newly formed collagen fibers are an indicator of healthy periodontal tissue.

The morphometric analysis was performed from the identification and quantification of newly formed collagen in 7 samples of each group by calculating the mean area fraction. The representative images were taken at 400X magnification using a digital camera attached to the inverted microscope. The computerized image analyzer program (ImageJ 1, 34 NIH, USA) was used to compute the mean area fraction.

#### Measurement of inflammatory cytokine

Levels of interleukin-1β (IL-1β) were identified using ELISA kits (Elabscience, USA. CAT No. E-HSEL-R0002, Detection Range; 0.31–20 pg/mL) in compliance with the guidelines provided by the manufacturer.

Frozen bone tissues were cleaned using ice-cold PBS (0.01M, pH = 7.4) and crushed into tiny pieces then weighed and homogenized in PBS buffer supplemented with detergent (0.1–1% Triton X-100) and protease inhibitors using a glass homogenizer on ice. Homogenates are centrifuged for 10–15 min at 5000×g at 2–8 ℃ to extract the supernatant (contains IL-1β). protein concentration was measured with Bradford assay. Aliquote supernatants were stored at –80 °C until ELISA. Cytokines concentrations were assessed and measured at optical density. Absorbance was read at 450 nm wavelength. ELISA assays were performed at Nawah scientific center, Cairo, Egypt.

#### Statistical analysis

To determine that the data are normally distributed, Kolmogorov–Smirnov normality test was used. The Bonferroni post hoc test and parametric ANOVA were used to evaluate the group differences. ANOVA was used to ascertain whether there were statistically significant differences between the newly formed area fractions per field and for IL-1β cytokines levels among groups. *p* values < 0.05 were considered significant using SPSS program, version 17, (IBM Corporation, Somers, New York, USA). The mean ± SD was used to represent the data.

## Results

### Characterization of BM-MSCs exosomes

**Fig. 1:**
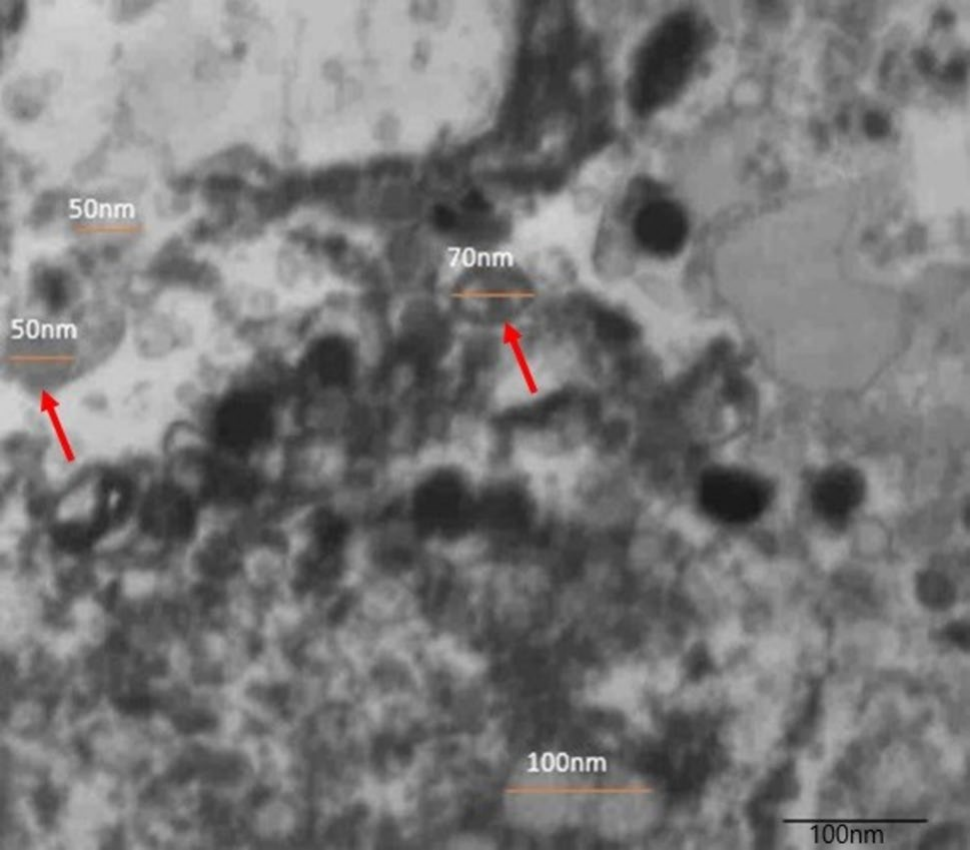
TEM photomicrograph of exosomes with ovoid shape, variable sizes, electron dense periphery and electron lucent center (arrows)

With transmission electron microscope multiple ovoid shape, variable sizes exosomes with electron dense periphery and electron lucent center were detected (Fig. 1).

### Characterization of curcumin nanoparticle loaded on exosomes

**Fig. 2:**
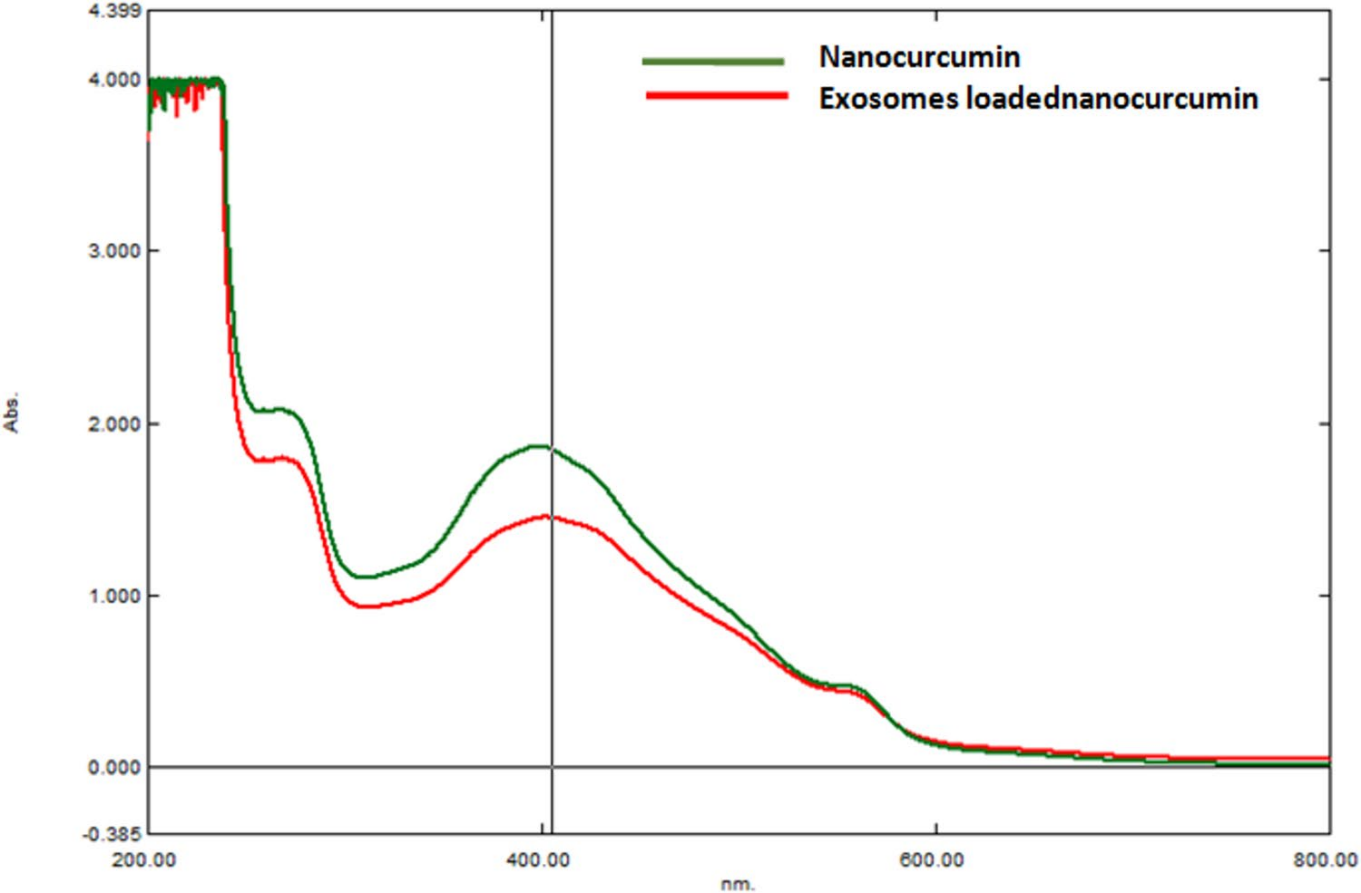
UV–visible spectrum showing a characteristic peak of nanocurcumin at 404 nm with different absorbance after loaded on exosomes

The absorbance of NCUR was measured using a UV–visible spectrophotometer at 404 nm. The results showed that the absorbance of NCUR without loading on exosomes was higher than the NCUR loaded on exosomes (Fig. 2).

### Histological results

#### Group I (Control)

**Fig. 3:**
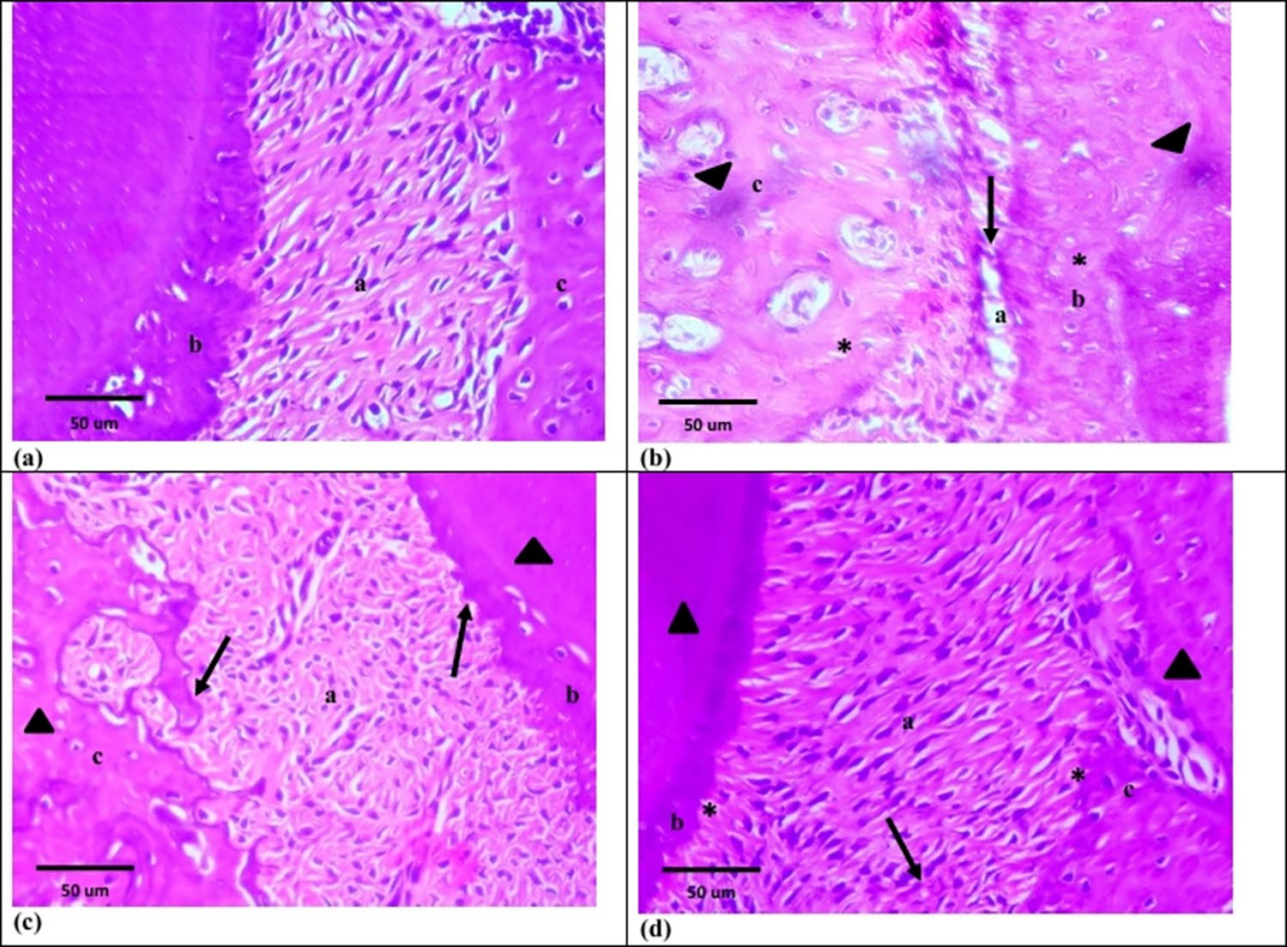
Photomicrographs of H&E sections at 2 weeks. **a **Group I showing: normal periodontal tissues; periodontal ligament (a), cementum (b) and alveolar bone (c). **b **Group II showing: wide areas of degeneration in periodontal ligament (a) as well as apparent reduction in its width (arrow). Both cementum (b) and alveolar bone (c) show different stainability (arrow heads) and few empty lacunae (asterisks). **c **Group III showing: disorganized dense periodontal ligament fibers (a). Both cementum (b) and alveolar bone (c) show consistent stainability (arrow heads) and irregular outlines (arrows). **d **Group IV showing: periodontal ligament fibers with oblique direction in some areas (a) and disorganized arrangement in other areas (arrows). Both cementum (b) and alveolar bone (c) show consistent stainability (arrow heads) and irregular outlines (asterisks)

**Fig. 4:**
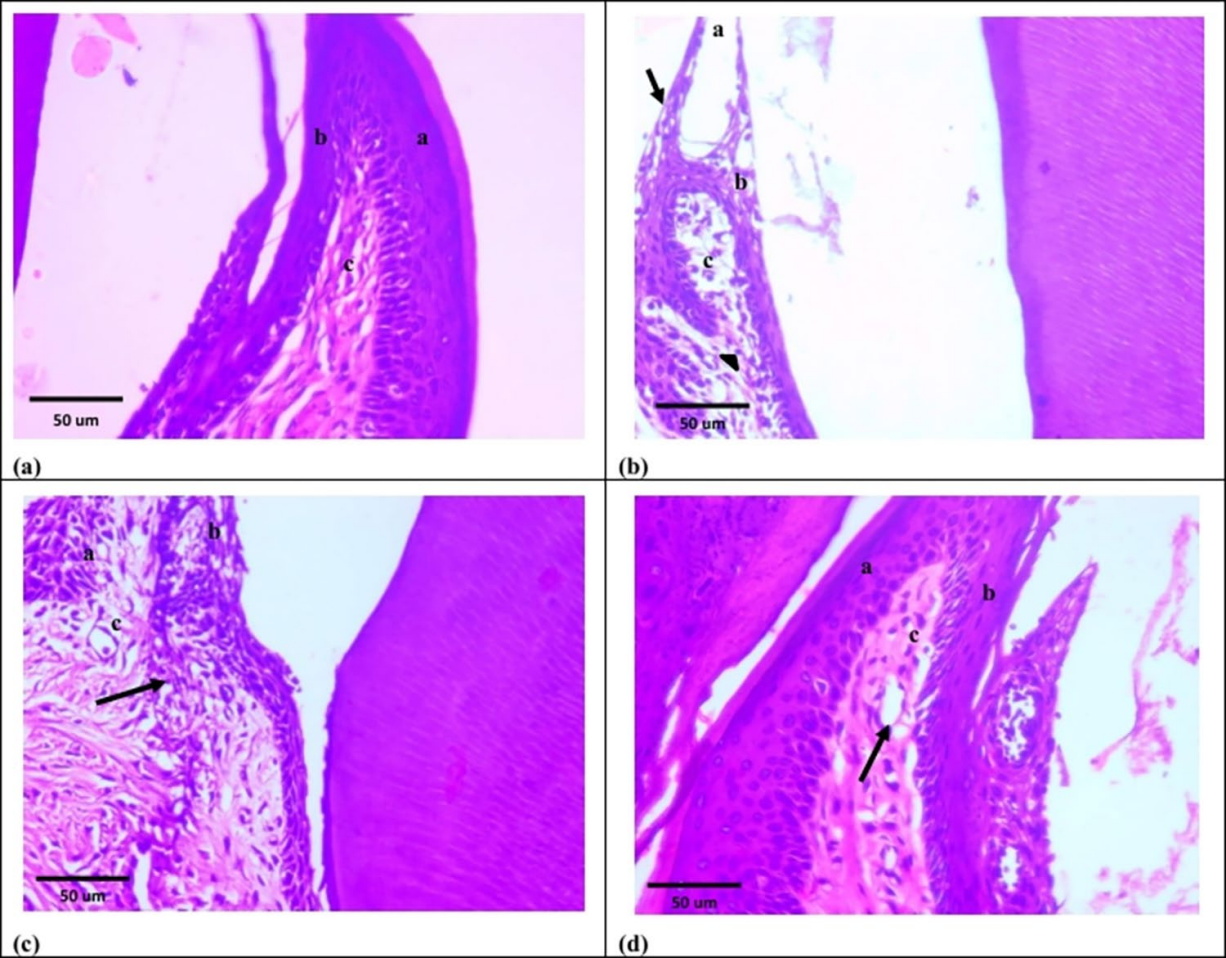
Photomicrographs of H&E sections at 2 weeks. **a **Group I showing: free gingiva covered by keratinized stratified squamous epithelium (a). Sulcular epithelium lined by non-keratinized stratified squamous epithelium (b). Lamina propria with dense connective tissue (c). **b **Group II showing: loss of keratin layer (arrow) and areas of degeneration in free gingiva (a). Sulcular epithelium illustrates areas of degeneration (b). The underlying lamina propria explores few collagen fiber bundles (arrow heads) and areas of degeneration (c). **c **Group III showing: areas of degeneration in epithelium of free gingiva (a). Sulcular epithelium shows small areas of degeneration (b). The lamina propria (c) explores disorganized collagen fiber bundles (arrow). **d **Group IV showing: keratinized and non-keratinized stratified squamous epithelium in free gingiva (a) and sulcular epithelium (b), respectively. Dense lamina propria (c) with visibly small areas of degeneration (arrow)

**Fig. 5:**
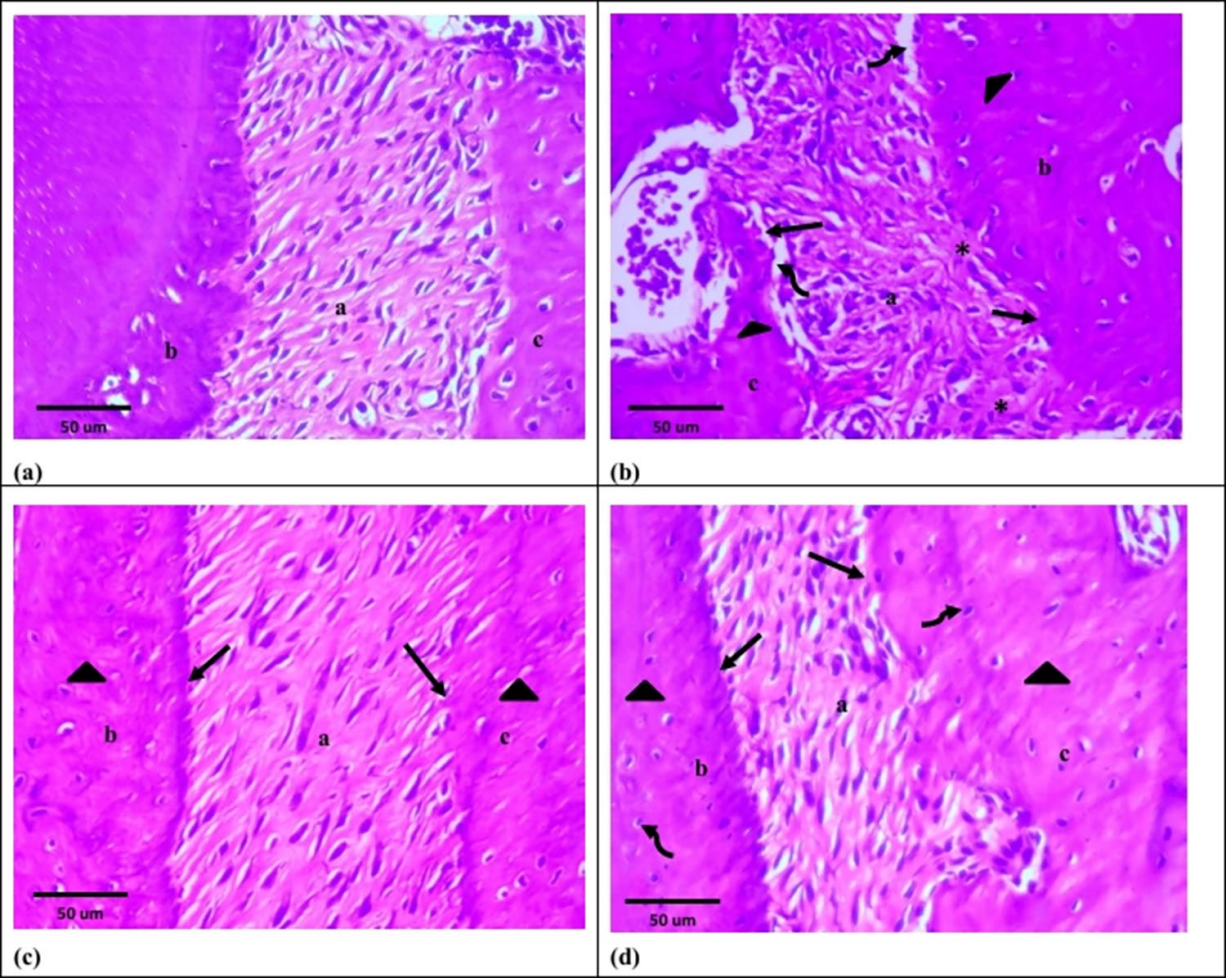
Photomicrographs of H&E sections at 4 weeks. **a **Group I showing: normal periodontal tissues; periodontal ligament (a), cementum (b) and alveolar bone (c). **b **Group V showing: disorganized periodontal ligament fibers (a) with some areas of hyaline degeneration (asterisks). Cementum (b) alveolar bone (c) showed different stainability (arrow heads), irregular outlines (arrows) and loss of Sharpey’s fibers (curved arrows). **c **Group VI showing: dense periodontal ligament fiber bundles with oblique direction (a). Both cementum (b) and alveolar bone (c) showed consistent stainability (arrow heads) and regular outlines with attached Sharpey’s fibers (arrows). **d **Group VII showing: dense periodontal ligament fiber bundles with oblique direction and wavy course (a). Both cementum (b) and alveolar bone (c) showed consistent stainability (arrow heads), regular outlines with attached Sharpey’s fibers (arrows), normal cementocytes and osteocytes (curved arrows)

**Fig. 6:**
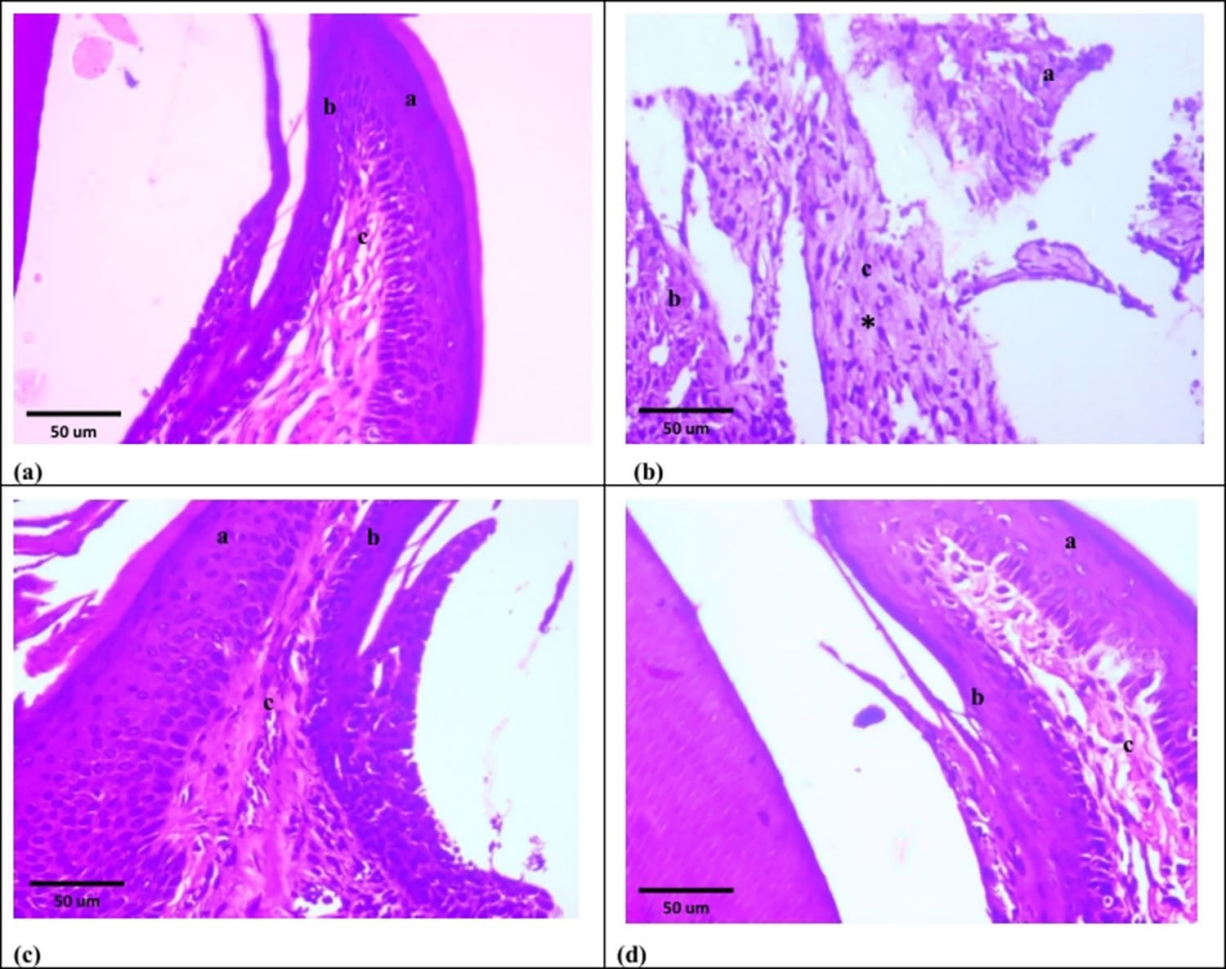
Photomicrographs of H&E sections at 4 weeks. **(a) **Group I showing: free gingiva covered by keratinized stratified squamous epithelium (a). Sulcular epithelium lined by non-keratinized stratified squamous epithelium (b). Lamina propria with dense connective tissue (c). **b **Group V showing: both sulcular epithelium (a) and epithelium of free gingiva (b) are detached from underlying lamina propria (c) which illustrates areas of hyaline degeneration (asterisk). **c **Group VI showing: free gingiva covered by keratinized stratified squamous epithelium (a). Sulcular epithelium lined by non-keratinized stratified squamous epithelium (b). Dense lamina propria (c). **d **Group VII showing: keratinized stratified squamous epithelium covering the free gingiva (a). Non-keratinized stratified squamous epithelium lines sulcular epithelium (b). Dense lamina propria with interlacing collagen fiber bundles (c)

Group I showed the normal structure of periodontium and gingiva. Periodontal ligament fibers were densely packed, obliquely arranged, wavy course and overlying fibroblasts. Both cementum and alveolar bone had normal stain, and their outlines were regular with presence of Sharpey’s fibers. Cementoblasts and cuboidal osteoblasts were over laying cementoid and osteoid layers, respectively. Normal cementocytes and osteocytes were detected (Figs. 3a, 5a). The free gingiva was covered by keratinized stratified squamous epithelium, while sulcular epithelium showed non-keratinized stratified squamous epithelium. The underlying lamina propria illustrated dense connective tissue with interlacing collagen fiber bundles and apparently few inflammatory cells (Figs. 4a, 6a).

### Group II (Periodontitis 2 weeks)

Group II illustrated apparent reduction in periodontal ligament width with sparse fibers, some cleaved bundles and apparently wide areas of degeneration. Some fibroblasts had nuclei with pyknotic and hyperchromatic appearance. Cementum and alveolar bone showed different stainability and obviously few empty lacunae. Absence of cementoid and osteoid layers were observed. Flat cementoblasts and obviously few osteoblasts were detected. However, in both tissues Sharpey’s fibers were attached (Fig. 3b). The epithelium of free gingiva revealed loss of keratin layer and areas of degeneration. Sulcular epithelium showed areas of degeneration and loss of basement membrane integrity. The underlying lamina propria explored noticeably few collagen bundles, degenerated areas, fibroblasts with pyknotic nuclei and some inflammatory cells (Fig. 4b).

### Group III (Periodontitis + exosomes 2 weeks)

Group III revealed disorganized dense periodontal ligament fibers and obviously small hyaline degeneration areas. Some fibroblasts illustrated pyknotic and hyperchromatic nuclei. Both cementum and alveolar bone had consistent staining, but their outlines were irregular. Reversal lines were detected in the alveolar bone. Strikingly few cementocytes and osteocytes showed pyknotic nuclei. Noticeably thin disrupted cementoid and osteoid layers with markedly few overlying cementoblasts and osteoblasts were observed (Fig. 3c). The epithelium of free gingiva revealed areas of degeneration and some cells showed hyperchromatic and pleomorphic nuclei. Sulcular epithelium showed apparently small areas of degeneration. The lamina propria explored disorganized collagen fiber bundles, obviously small areas of hyaline degeneration, fibroblasts with pyknotic nuclei and some inflammatory cells (Fig. 4c).

### Group IV (Periodontitis + loaded exosomes 2 weeks)

Group IV illustrated periodontal ligament fibers with oblique direction in some areas and disorganized arrangement in other areas. Most of fibroblasts were spindle shaped but obviously few showed pyknotic nuclei. Cementum and alveolar bone were irregular but had consistent staining. Obviously, few osteocytes showed pyknotic nuclei. Both cementoid and osteoid layers as well as the overlying cementoblasts and osteoblasts were hardly detected (Fig. 3d). Keratinized and non-keratinized stratified squamous epithelium were observed in free gingiva and sulcular epithelium, respectively. Dense lamina propria was detected with visibly small areas of degeneration and few inflammatory cells (Fig. 4d).

### Group V (Periodontitis 4 weeks)

Group V explored disorganized periodontal ligament fibers with some hyaline degeneration areas as well as inflammatory cells infiltration. Pyknosis and hyperchromatism were observed in most fibroblasts’ nuclei. Cementum and alveolar bone were irregular, showed difference in staining, apparently few empty lacunae as well as degenerated cementocytes and osteocytes, respectively. Loss of Sharpey’s fibers were observed. Absence of cementoid and osteoid layers were detected. Cementoblasts and osteoblasts showed pyknotic as well as hyperchromatic nuclei (Fig. 5b). Both sulcular epithelium and epithelium of free gingiva were detached from underlying lamina propria which illustrated areas of hyaline degeneration, fibroblasts with pyknotic nuclei and inflammatory cells infiltration (Fig. 6b).

### Group VI (Periodontitis + exosomes 4 weeks)

Group VI explored dense periodontal ligament fiber bundles with oblique direction. Most of fibroblasts were spindle shaped; however, perceptibly few showed pyknotic nuclei. Cementum and alveolar bone were regular, showed consistent staining and Sharpey’s fibers. Resting lines were detected in the alveolar bone. Both cementocytes and osteocytes appeared normal. Ostensibly few flat cementoblasts and osteoblasts were overlying cementoid and discontinued osteoid layers, respectively (Fig. 5c). The free gingiva was covered by keratinized stratified squamous epithelium. Sulcular epithelium showed non-keratinized stratified squamous epithelium. Dense lamina propria was detected with apparently few inflammatory cells (Fig. 6c).

### Group VII (Periodontitis + loaded exosomes 4 weeks)

Group VII explored dense periodontal ligament fiber bundles with oblique direction and wavy course. Fusiform fibroblasts were detected. Both cementum and alveolar bone were regular with consistent staining, normal cementocytes and osteocytes as well as Sharpey’s fibers. Alveolar bone had obvious resting lines. Plumped cementoblasts and osteoblasts were detected over cementoid and deceptively thin osteoid layers, respectively (Fig. 5d). Keratinized stratified squamous epithelium was covering the free gingiva. Non-keratinized stratified squamous epithelium was detected in sulcular epithelium. Dense lamina propria with interlacing collagen fiber bundles and markedly few inflammatory cells were detected (Fig. 6d).

### Results of Masson's trichrome

New collagen was detected by blue color, while old collagen was detected by red color.

**Fig. 7:**
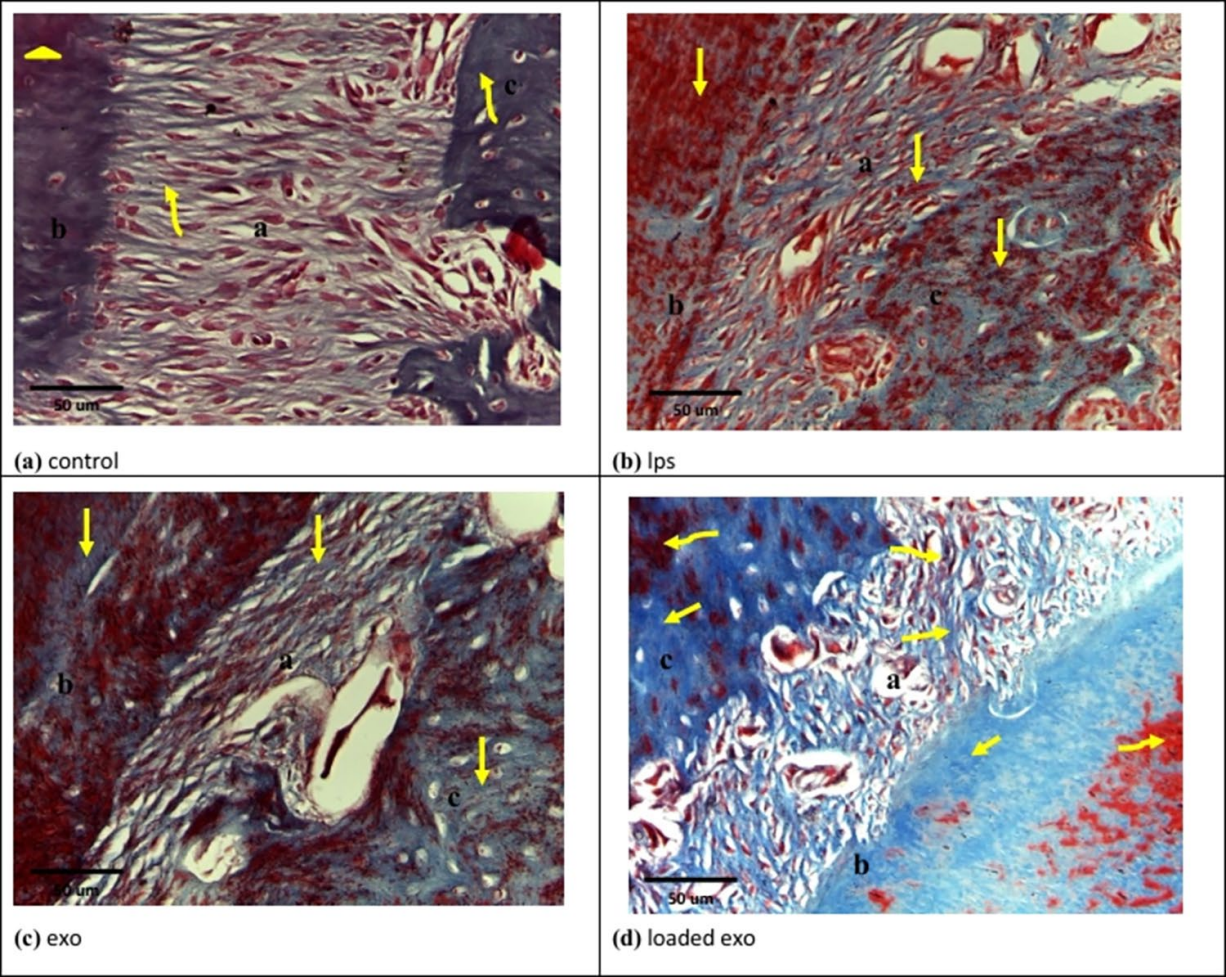
Photomicrographs of Masson’s trichrome sections at 2 weeks. **a **Group I showing: periodontal ligament fibers (a) and alveolar bone (c) with apparently large areas of newly formed collagen (curved arrows). Cementum (b) shows old collagen mainly (arrow head). **b **Group II showing: wide areas of old collagen (arrows) in periodontal ligament fibers (a), cementum (b) and alveolar bone (c). **c **Group III showing: new collagen (arrows) in some areas of periodontal ligament fibers (a), cementum (b) and alveolar bone (c). **d **Group IV showing: periodontal ligament fibers (a), cementum (b) and alveolar bone(c) with large areas of newly formed collagen (arrows). The old collagen was restricted to small areas (curved arrows)

**Fig. 8:**
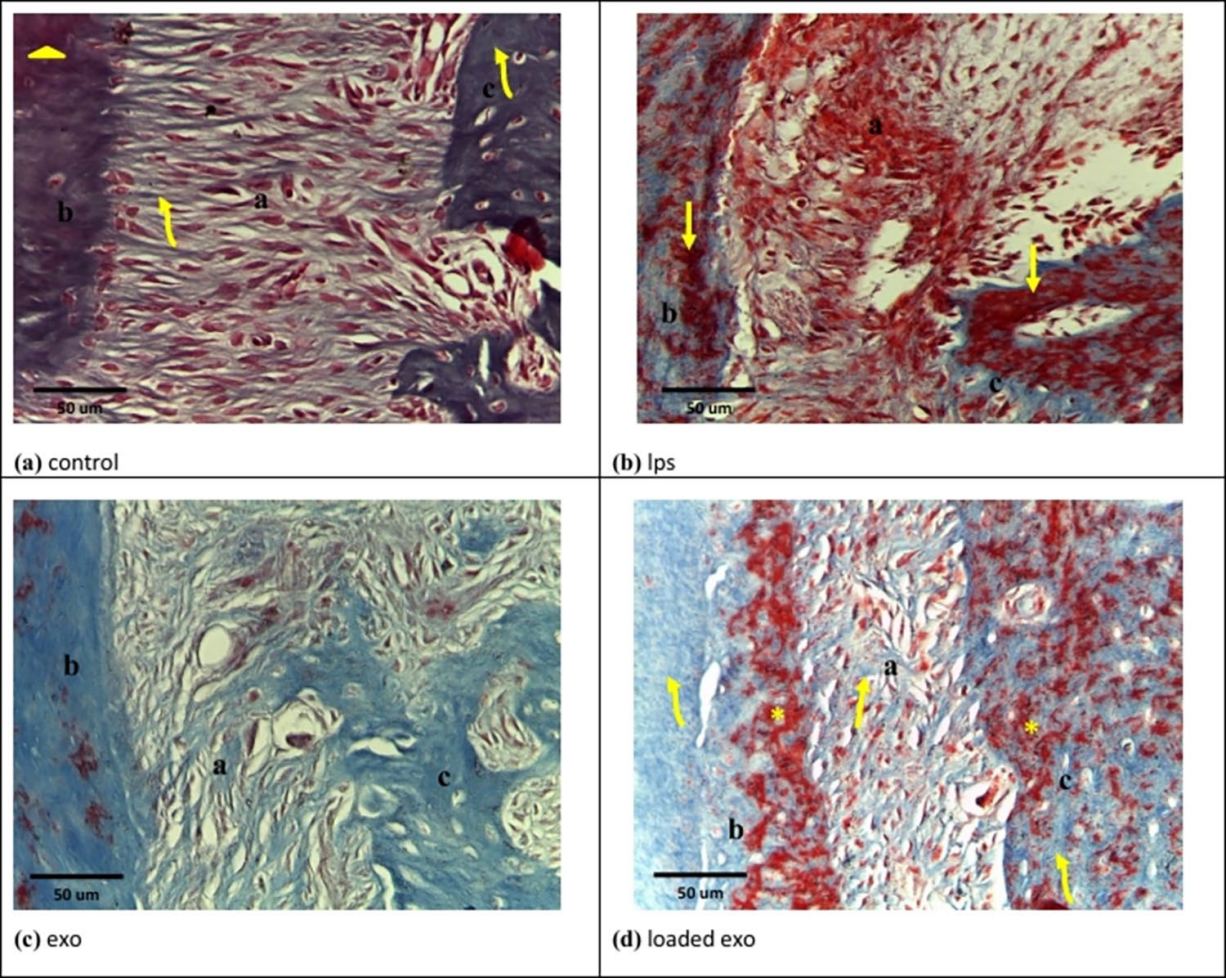
Photomicrographs of Masson’s trichrome sections at 4 weeks. **a **Group I showing: periodontal ligament fibers (a) and alveolar bone (c) with large areas of newly formed collagen (curved arrows). Cementum (b) shows old collagen mainly (arrow head). **b **Group V showing: mainly old collagen in periodontal ligament fibers (a) and wide areas of old collagen (arrows) in cementum (b) and alveolar bone (c). **c **Group VI showing: only newly formed collagen in periodontal ligament fibers (a), cementum (b) and alveolar bone (c). **d **Group VII showing: periodontal ligament fibers (a) with wide areas of newly formed collagen (arrow). Cementum (b) showed intermingled areas of old collagen (asterisk) and new collagen (curved arrow). Alveolar bone (c) showed wide areas of new collagen (curved arrow), while old collagen was detected in apparently few areas (asterisk)

Group I illustrated obviously wide areas of new collagen in periodontal ligament fibers and alveolar bone; however, cementum exhibited old collagen mainly (Figs. 7a, 8a). On the other hand, Groups II and V illustrated obviously wide areas of old collagen in periodontium (Figs. 7b, 8b). Group III demonstrated new collagen in some areas (Fig. 7c).

Group IV showed wide areas of new collagen in periodontal ligament fibers, cementum and alveolar bone; however, old collagen was restricted to obviously small areas (Fig. 7d). Group VI demonstrated only newly formed collagen (Fig. 8c).

Group VII showed obviously wide areas of new collagen in periodontal ligament and alveolar bone. Cementum showed intermingled areas of new and old collagen (Fig. 8d).

### Statistical analysis results

#### Newly formed collagen means area fraction

**Fig. 9:**
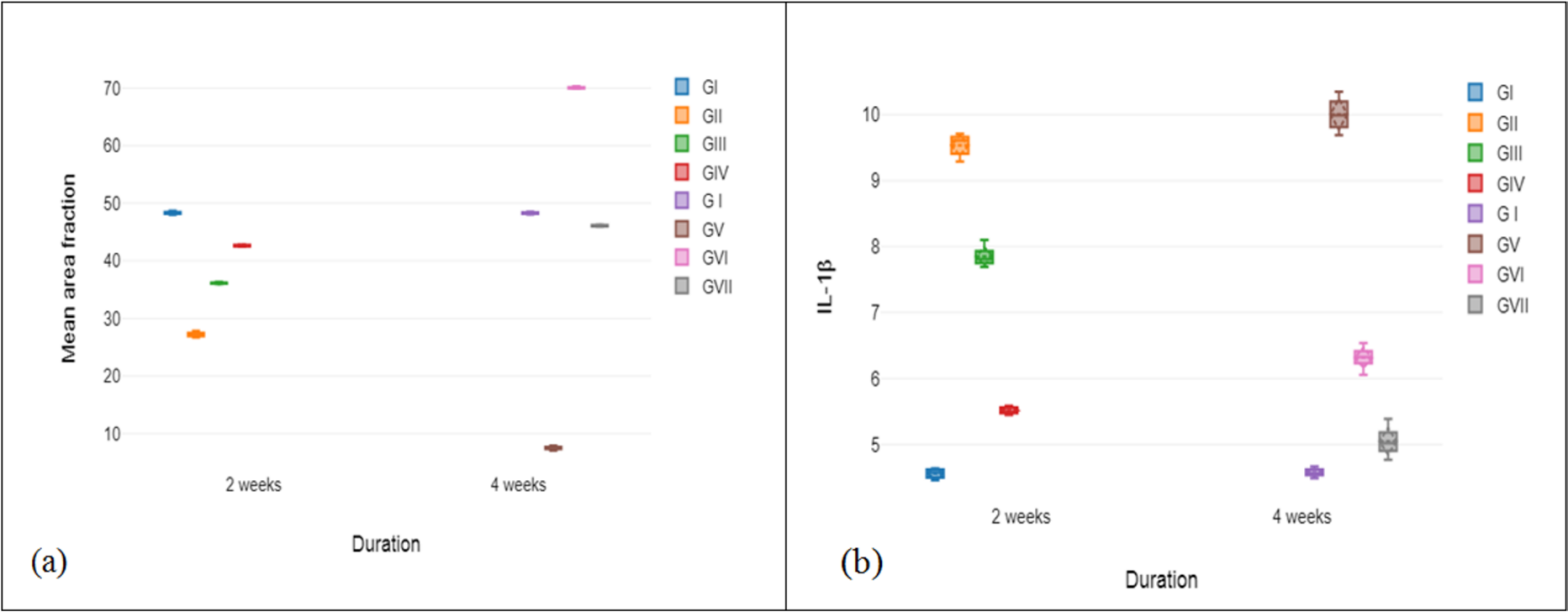
Box plot represents means and SD values in all studied groups (**a**) newly formed collagen mean area fraction in all studied groups. **b** IL-1β cytokine

With regard to histomorphometry, all treatment groups have statistically significant differences with respect to the control (*p* < 0.001). In the measurement of new collagen mean area fraction, group VI has the highest mean, followed by group VII, group IV and group III. Group V has the lowest new collagen mean area fraction. Group VII presents little difference with group I (Table 1, Fig. 9a).

**Table 1:** Mean ± SD values, results of ANOVA as well as Bonferroni post hoc tests for the comparison between different studied groups regarding newly formed collagen mean area fraction

Duration	Groups	N	Mean ± Std µm^2^	***ANOVA*** F		*p* value
2 Weeks	GI	7	48.31 ± 0.25	2–4 Weeks	4600.81	< 0.001^*^
	GII	7	27.25 ± 0.43^acde^			
	GIII	7	36.08 ± 0.15^abdf^	Groups	60,439.82	< 0.001^*^
	GIV	7	42.61 ± 0.16^abcg^			
4 Weeks	GI	7	48.27 ± 0.19			
	GV	7	7.5 ± 0.3^af^	2–4 Weeks x Groups	29,130.86	< 0.001^*^
	GVI	7	70.09 ± 0.16^ae^			
	GVII	7	46.06 ± 0.16^aef^			

According to *Bonferroni* post hoc test, indication of superscript letters is; a: significant difference with GI; b: significant difference with GII; c: significant difference with GIII; d: significant difference with GIV; e: significant difference with GV; f: significant difference with GVI; g: significant difference with GVII

^*^Highly significant at *p* value < 0.001

#### IL-1β

All treated groups have statistically significant differences with respect to the control (*p* < 0.001). Group V has the greatest IL-1β mean. On the other hand, groups VII, IV and VI show lower means, respectively (Table 2, Fig. 9b).

**Table 2:** Mean ± SD values, results of ANOVA as well as Bonferroni post hoc tests for the comparison between different studied groups regarding IL-1β

Duration	Groups	N	Mean ± Std Pg/mL	***ANOVA*** F		*p* value
2 Weeks	GI	7	4.56 ± 0.07	2–4 Weeks	81.15	< 0.001^*^
	GII	7	9.54 ± 0.17^acde^			
	GIII	7	7.85 ± 0.14^abdf^	Groups	3056.26	< 0.001^*^
	GIV	7	5.52 ± 0.05^abcg^			
4 Weeks	GI	7	4.58 ± 0.06			
	GV	7	10.01 ± 0.25^af^	2–4 Weeks x Groups	104.91	< 0.001^*^
	GVI	7	6.32 ± 0.16^ae^			
	GVII	7	5.05 ± 0.22^aef^			

According to *Bonferroni* post hoc test, indication of superscript letters is; a: significant difference with GI; b: significant difference with GII; c: significant difference with GIII; d: significant difference with GIV; e: significant difference with GV; f: significant difference with GVI; g: significant difference with GVII

^*^Highly significant at *p* value < 0.001

## Discussion

Periodontitis is one of the main causes of incurable degradation of the supporting periodontium [1]. Escherichia coli LPS was used in our investigation as it exhibits greater endotoxic intensity. It also triggers more inflammatory signaling than Porphyromonas gingivalis [20]. Meanwhile, Wister albino rat strain was chosen, because it is most commonly utilized in experimental studies of periodontitis [21].

In this investigation, the deteriorations observed within group V were more pronounced compared to group II. Elevated expression of nuclear factor kappa-B and activator protein1 is a consequence of excessive release of inflammatory mediators, for instance IL-1β in LPS-induced periodontitis. This has been identified as a main stimulus for damage of supporting apparatus and bone resorption [21]. The breakdown of extracellular matrix constituents is also associated with the production of matrix metalloproteinases, which may end in destruction of periodontal attachment and subsequent bone resorption [22]. Overabundance of reactive oxygen species (ROS) together with proteases is yet another hallmark of periodontitis. Increased ROS level causes the oxidant load to rise and the antioxidant capacity to either remain unchanged or decrease. As a result, the impacted tissues experience oxidative stress, which ultimately causes the loss of teeth and the supporting tissues. Intracellularly, ROS harm biomolecules and cell membranes initiating cellular disintegration [23].

Exosomes have the potentiality to promote healing of periodontitis and other bone defects [24]. In the herein study, exosomes were derived from 4-week-old rat BM-MSCs. This goes in parallel with Xu et al. [25] who postulated that rats injected with exosomes from the aforementioned age BM-MSCs would show improved collagen synthesis and bone deposition in fractured femur. The used dose in this investigation (200 μg BMSC-Exo) was proved to demonstrate the best therapeutic effectiveness in restoration of calvaria defects [26]. BMSC-Exo were delivered by subgingival injection, because it is less invasive and accessible approach. Besides, local delivery permits only limited exposure of exosomes at the required location. Therefore, they undergo rapid absorption by the cells in the conveyed location, hence lessening a strayed target influence, also minimizing curing expenses [11, 27].

The present research revealed cementoid and osteoid layers with overlying cementoblasts and osteoblasts. Cementum and alveolar bone revealed irregular outlines in group III, while both tissues appeared with regular borders and attached Sharpey’s fibers in group VI. These results were supported by Lin et al., Wei et al. and Zhang et al. [12, 23, 28] who proved that human exfoliated deciduous teeth derived exosomes (SHED-Exo), periodontal ligament stem cell derived exosomes (PDLSC-Exo) and MSC exosomes markedly repaired loss of alveolar bone and enabled production of new bone in mouse and rat periodontitis, respectively. Furthermore, osteoblast-like cells and new bone formation were observed in rats and mice treated with MSCs exosomes in calvaria defect model [29–32]. Further investigation declared wider area of newly formed bone and osteoblast-like cells in 4 weeks than 2-week group following implantation of MSCs-Exo in calvaria defect of rat model [32]. One proposed explanation is that MSC exosomes promote bone regeneration through complex mechanisms by stimulation of osteogenesis and angiogenesis, raising survival, proliferation, migration of osteoblasts and hindering osteoclastic maturation [33, 34]. This favorable action of MSCs-Exo in bone renewal may be related to immediate delivery of their contents and concurrent control of downstream signals in the targeted cells, in addition to up-regulating anti-inflammatory mediators as IL-10 [26, 35]. Exosomes may additionally influence bone tissue regeneration by transmitting miRNAs. Elevated levels of miRNAs promote the expression of Runx2 and alkaline phosphatase, which triggers the Wnt signaling pathway and allowing formation of osteoblasts [9, 32].

The herein study showed improved histological features from group III to group VI regarding periodontal ligament fibers and gingiva. In agreement with this point, SHED-exosomes and BMSC-Exo enabled periodontal regeneration with reorganization and repair of gingival fibers in mouse and rat periodontitis, respectively [24, 36]. In addition, LPS-stimulated fibroblasts treated in vitro with BM-MSC exosomes reported reconstructed intact cell membrane and evident cell organelles [37]. Likewise, MSC exosomes implanted in periodontal intrabony defects in immunocompetent rats presented improvement in regeneration and orientation of periodontal fibers, gingival tissue and bone 4-week postimplantation compared to 2-week group [38]. A proposed explanation suggested that despite exosomes’ quick discharge, their impact seemed to last and extend for at least 4 weeks as evident by accelerated bone and periodontal tissue regeneration. Endogenous reparative processes may be initiated, also a sustained regenerative process may be set off by a transient exposure to MSC exosomes [1]. The almost normal structural features of periodontal tissue observed in BMSC-Exo group could be referred to rise in growth factors and genes linked to periodontal matrix synthesis which facilitate fibroblasts adhesion and migration. This is hypothesized to aid in periodontal regeneration by attracting periodontal fibroblasts to produce new periodontal tissue [39]. MSC exosomes also promote periodontal restoration by promoting the viability of cell, differentiation, migration, proliferation and matrix production to establish superior periodontal attachment [1]. It was also suggested that the anti-inflammatory and antioxidant criteria of BMSC-Exo might be contributed to their regenerative potential [40]. Apparently, few inflammatory cells were detected in groups III and VI in the lamina propria of the attached gingiva. Alike, Kim & Zhang et al. [27, 28] identified reduced number of inflammatory cell infiltration of attached gingiva in BMSC-Exo treated periodontitis in rats and mice. This finding could be implicated to the anti-inflammatory function of exosomes in periodontal disease models.

Nanotechnology is becoming more significant in the field of dental medicine, specifically in periodontal therapy by stimulating cellular proliferation and periodontal repair [41]. Dissolved NCUR were used in this research to enhance their physicochemical characteristics by reducing particle size and forming a high-energy amorphous state that improves medication release and solubility [5]. Numerous investigations have documented the repair of alveolar bone loss by administration of curcumin or NCUR in induced periodontitis of rats [5, 21, 23, 42]. In addition, in vitro experiments on PDLSCs subjected to curcumin verified elevated rate of osteoblastic differentiation [43]. It was determined that curcumin increases the amount of collagen, triggers fibroblastic proliferation and represses inflammatory cells within periodontal defects [15, 23]. Compatible outcomes were notified when curcumin gel was imbedded on the back skin of injured rats [44]. It is conceivable to suggest that curcumin's regenerative and anti-inflammatory properties stem from its capacity to boost antioxidant activity through modulation of glutathione peroxidase, catalase, or superoxide dismutase. This subsequently lowers oxidative damage within the afflicted tissues like periodontium [45, 46]. It was further documented that curcumin inhibits spreading of osteoclast precursors, which in turn hinders the development of matured osteoclasts. Therefore, this offers suggestions for enhancing its treatment approaches to prevent loss of bone [47].

An unconventional method for administering curcumin is to apply extracellular vesicles as carriage agent [48]. In this work, we used MSCs-Exo as carriers for NCUR, since most exosomes are lipid-enriched and hydrophobic, they have the potential to transport compounds with medicinal value such as curcumin [10, 48]. Besides, exosomes have directing definite properties and possess inherent biological impacts on the targeted cells [48]. Furthermore, sonication was the method of choice for curcumin loading on MSCs-Exo in this study. It was proved that sonication had superior loading efficacy comparable to different techniques for loading catalase into exosomes [49]. Meanwhile, when sonication was used instead of agitating approach, the loading effectiveness of paclitaxel on exosomes increased by four times. The authors attributed the enhanced loading capability to the short-term pores that are produced during sonication which enable the medication to effectively permeate into the exosomes [50]. Moreover, we have to report here that the selectivity of NCUR-loaded exosomes technology delivers an appropriate option for curcumin and other natural anti-inflammatory substances to attack the inflammatory cells and to get control of undesired outside-target reactions which confine their utility [10]. In addition, curcumin may become more soluble, stable, and bioavailable when it is incorporated into exosomes [51, 52]. Over and above, curcumin promotes the regenerative ability of MSCs via reducing oxidative stress and enhancing their secretion of growth factors [53, 54].

Our research revealed improved periodontal status by presence of dense periodontal ligament fiber bundles with oblique direction. Cementum and alveolar bone showed defined outlines with thin cementoid and osteoid layers in 4 weeks treated group (VII). Almost normal gingival structure was also observed. We supposed that NCUR and MSCs-Exo act synergistically to provide better histological results. This could be attributed to their anti-inflammatory action via up-regulating IL-10 levels, arresting ROS, anti-apoptotic proteins and inflammatory cytokines like IL-1β. The prolonged and continuous drug delivery pattern to the specified areas without altering the biological functions of normal cells provides another rationale [18, 48, 55]. Moreover, according to other researchers, curcumin-loaded hydrogels displayed encouraging tissue regeneration potential through stimulating collagen deposition, fibroblast and keratinocyte migration and proliferation to the damaged site [56].

Newly formed collagen means area fraction results revealed highest values in curcumin loaded and exosomes treated groups (VI, VII, IV, III). Lowest values were expressed in induced periodontitis groups (V and II). These data confirm to studies which investigated the effect of human exfoliated deciduous teeth-derived exosomes and human MSC exosomes in rat model periodontitis. Masson stain revealed that the exosome-treated groups featured newly organized gingival and periodontal fibers which were restricted in the periodontitis group [1, 24]. In accordance, trichrome staining confirmed that mesenchymal stromal cells exosomes generated from adipose tissue enhanced the production and deposition of collagen fibers in tooth sockets [57]. As well, when NCUR were applied locally to detect rats' periodontal healing, the collagen content increased significantly compared to the untreated group using trichrome stain [58]. It is feasible to suggest that matrix metalloproteinases released in periodontitis may degrade collagen, break down extracellular matrix proteins, and disrupt the activity of periodontal ligament fibroblasts [59]. On the other hand, exosomes are thought to assist periodontal renewal by facilitating differentiation and proliferation of fibroblasts for synthesis of new collagen fibers [1]. It is necessary to mention that during periodontal repair the primary type of collagen formed is type III immature collagen [60]. Meanwhile, researches proved that curcumin-treated animals conveyed high collagen percent and maturation together with increased extracellular density. This may be attributed to its anti-inflammatory action, plus its capability to enhance collagen deposition and cellular proliferation during inflammatory resolution [5, 61].

ELISA results proved that the greatest mean of IL-1β was expressed in group V and decreased in curcumin loaded and exosomes treated groups (VII, IV and VI). These findings were in close agreement with [11, 48, 62–65]. It was speculated that LPS show upregulation of the pro-inflammatory mediator IL-1β, which permits ROS production and triggers neutrophils chemotaxis and exocytosis, subsequently provoking periodontal inflammation and tissue breakdown [64, 66]. However, BMSC-Exo display an antioxidant effect, besides their capability to down-regulate pro-inflammatory mediators via inhibition of inflammatory pathways mediated by IL-1β [67, 68]. Concomitantly, one of the power sources behind the anti-inflammatory behavior of curcumin occurs through eradication of the produced free radicals as well as attenuation of IL-1β expression [69].

Limitations of this study include exclusion of female rats due to hormonal changes and extending the experiment duration to avoid rats’ senility.

## Conclusions

To sum up, this study represents a basic investigation into the exosomal NCUR drug delivery system and may mark the beginning of a potent new local delivery approach. The use of NCUR-loaded BMSC-Exo may benefit from the functional characteristics of exosomes, nanoparticles, and curcumin in a powerful yet secure targeting method to treat a range of inflammatory-related disorders in a time-dependent manner.

## Data Availability

All data and material are available upon request.

## Abbreviations

BMSC-Exo: Bone marrow mesenchymal stem cells.
H&E: Hematoxylin and eosin.
hPDLSCs: Human periodontal ligament stem cells.
IL-1β: Interleukin 1 beta.
LPS: Lipopolysaccharides.
NCUR: Nanocurcumin.
PBS: Phosphate buffer saline.
PDLSC-Exo: Periodontal ligament stem cell derived exosomes.
ROS: Reactive oxygen species.
SHED-Exo: Human exfoliated deciduous teeth derived exosomes
